# Genome-Wide Association Mapping Identifies Novel Panicle Morphology Loci and Candidate Genes in Sorghum

**DOI:** 10.3389/fpls.2021.743838

**Published:** 2021-10-05

**Authors:** Lihua Wang, Hari D. Upadhyaya, Jian Zheng, Yanlong Liu, Shailesh Kumar Singh, C. L. L. Gowda, Rajendra Kumar, Yongqun Zhu, Yi-Hong Wang, Jieqin Li

**Affiliations:** ^1^College of Agriculture, Anhui Science and Technology University, Chuzhou, China; ^2^Gene Bank, International Crops Research Institute for the Semi-Arid Tropics (ICRISAT), Patancheruvu, India; ^3^Division of Genetics, ICAR-Indian Agricultural Research Institute, New Delhi, India; ^4^Institute of Agricultural Resources and Environment, Sichuan Academy of Agricultural Sciences (SAAS), Chengdu, China; ^5^Department of Biology, University of Louisiana at Lafayette, Lafayette, LA, United States

**Keywords:** sorghum, panicle morphology, association mapping, mini core, candidate genes

## Abstract

Panicle morphology is an important trait in racial classification and can determine grain yield and other agronomic traits in sorghum. In this study, we performed association mapping of panicle length, panicle width, panicle compactness, and peduncle recurving in the sorghum mini core panel measured in multiple environments with 6,094,317 single nucleotide polymorphism (SNP) markers. We mapped one locus each on chromosomes 7 and 9 to recurving peduncles and eight loci for panicle length, panicle width, and panicle compactness. Because panicle length was positively correlated with panicle width, all loci for panicle length and width were colocalized. Among the eight loci, two each were on chromosomes 1, 2, and 6, and one each on chromosomes 8 and 10. The two loci on chromosome 2, i.e., *Pm 2-1* and *Pm 2-2*, were detected in 7 and 5 out of 11 testing environments, respectively. *Pm 2-2* colocalized with panicle compactness. Candidate genes were identified from both loci. The rice *Erect Panicle2* (*EP2*) ortholog was among the candidate genes in *Pm 2-2*. *EP2* regulates panicle erectness and panicle length in rice and encodes a novel plant-specific protein with unknown functions. The results of this study may facilitate the molecular identification of panicle morphology-related genes and the enhancement of yield and adaptation in sorghum.

## Introduction

The sorghum inflorescence consists of a single panicle with many racemes and is an important determinant of grain yield (Hmon et al., 2013). Sorghum panicles are more extensively branched than maize and rice (Vollbrecht et al., 2005; Brown et al., 2006) and vary significantly in number, length, and angle of primary branches as well as the three-dimensional shape, size, and distribution of the seed (Li et al., 2020), especially compared to other major cultivated cereal crops (Brown et al., 2006). Therefore, sorghum is an excellent model for studying panicle morphology in panicle-bearing grasses. Sorghum panicles may be compact or open up to 50 cm long and 30 cm wide (Doggett, 1988), and their morphology depends on the number and length of panicle branches and the number of aborted spikelets (Brown et al., 2006). The panicle morphology is an important criterion for the racial classification of sorghum. The compact panicle is typical of domesticated sorghum, especially elite high-yielding modern commercial varieties (Kimber, 2000; Brown et al., 2006; Dillon et al., 2007; OGTR, 2017), whereas undomesticated species are more likely to have open panicles (Harlan and de Wet, 1972). Plants with open or loose panicles are more likely to be small-seeded, reducing grain yield (Desmae et al., 2016). However, compact panicles are also more prone to infection/infestation by grain mold (Sharma et al., 2010), webworm [*Celama sorghiella* (Riley)] (Hobbs et al., 1979), head bug (*Calocoris angustatus* Leth.), and head caterpillar (*Helicoverpa armigera* Hb.) (Sharma et al., 1994). As a result, race guinea with loose panicles is more common in wet environments to prevent grain molding, and race durra with compact panicles is more common in dry environments (Harlan and de Wet, 1972; Doggett, 1988; Ayana and Bekele, 1998).

Despite its importance in yield and adaptation, the genetic control of panicle morphology is not fully understood. Approximately 300 panicle morphology-related quantitative trait loci (QTLs) have been cataloged by Mace et al. (2019) from previous studies. More recently, Girma et al. (2019) identified 15 regions across the sorghum genome associated with panicle compactness and shape, and Faye et al. (2019) identified 13 panicle compactness loci that colocalize with *a priori* candidate genes. Olatoye et al. (2020) also found a significant enrichment of QTL colocalized with grass panicle-related genes such as maize *Ramosa2* and rice *Aberrant Panicle Organization1* (*APO1*) and *TAWAWA1*, but many QTLs did not colocalize with panicle gene orthologs (Olatoye et al., 2020). They suggested that global panicle diversity in sorghum is largely controlled by oligogenic, epistatic, and pleiotropic variations in ancestral regulatory networks. Zhou et al. (2019) detected 35 unique SNPs associated with variation in panicle architecture using a semiautomated phenotyping pipeline called Toolkit for Inflorescence Measurement (TIM). They also found colocalization with previously mapped panicle-related loci and identified nine candidate genes.

The objective of this study was to identify QTL related to panicle morphology and recurving of peduncles and determine the candidate genes that regulate panicle morphology in sorghum using a genome-wide association study (GWAS) with phenotyping data on sorghum panicle length and width in 11 environments at International Crops Research Institute for the Semi-Arid Tropics (ICRISAT), India, panicle compactness in two environments in China, and 6,094,317 single nucleotide polymorphism (SNP) markers in the sorghum mini core (MC) collection panel (Upadhyaya et al., 2009).

## Materials and Methods

A total of 242 accessions of sorghum MC (Upadhyaya et al., 2009) were phenotyped in rainy and post-rainy seasons with or without irrigation at ICRISAT, Patancheru, India. The plants were grown in an alpha design with three replicates. Each single-row plot was 4 m long with a row spacing of 75 cm and plant spacing within a row of 10 cm. Ammonium phosphate (150 kg/ha) was applied before planting, and 100 kg/ha of urea was applied as a top dressing 3 weeks after planting. For the post-rainy season with irrigation, field plots were irrigated five times at equal intervals, each with 7 cm of water. Panicle length and width were measured in centimeters according to the International Board for Plant Genetic Resources IBPGR/ICRISAT (1993).

The MC panel was also grown in Tengqiao, Hainan, China (18°24′ N, 109°45′ E) in 2017 and 2020. All experiments used a completely randomized block design with three replicates. Before harvest, panicle pictures were taken and panicle compactness, length/width, and peduncle recurving were scored according to IBPGR/ICRISAT (1993). When panicles were scored as 1 = loose, 2 = semi-compact/semi-loose, and 3 = compact (Mohammed et al., 2015), the original IBPGR/ICRISAT codes of 1, 2, 3, 4, and 11 were converted to 1; 6 and 7 to 2; and 8, 9, 10, and 13 to 3. The coefficient of variation (CV) was calculated as the ratio between SD and mean. The broad-sense heritability was calculated using the R lme4 package.

The genome resequencing of 242 MC accessions and SNP development was performed as follows. The reference genome was the sorghum BTx623 (Paterson et al., 2009) version 3.1.1 (https://phytozome-next.jgi.doe.gov/info/Sbicolor_v3_1_1), which was also used to identify candidate genes. Sequencing reads were mapped to the reference genome using BWA-MEM version 0.7.17 (Li, 2013) and sorted by SAMtools version 1.10 (Li et al., 2009). Duplicate reads were removed using Picard version 2.0.1 (http://broadinstitute.github.io/picard/). The SAMtools flagstat was used to calculate the mapping percentage. Sequence variation detection and SNP calling were performed using the GATK version 4.17 function HaplotypeCaller and SelectVariants (McKenna et al., 2010). SNPs were called with parameters “QD <2.0, MQ <40.0, FS > 60, SOR > 3.0, MQRankSum < −12.5, ReadPosRankSum < −8.0.” SNPs were filtered with VCFtools version 1.16 (Li, 2013) using the parameters “max-missing 0.1, maf 0.05, maxDP 50, and minDP 10.” Only SNPs on chr1–chr10 were used. This produced 6,094,317 SNPs for the GWAS analysis. Population structure was analyzed using Admixture version 1.3 (Alexander et al., 2015). The number of clusters (k) in MC was set to 2–15. Admixture version 1.3 was run for each *k*-value, using 489,339 SNPs (Supplementary Figure 1). The optimal *k* was determined to be 10, as the CV (i.e., cross-validation) error was the lowest at *k* = 10. This *k*-value was used to generate the Q matrix used in the GWAS, as described below.

The GWAS and linkage disequilibrium (LD) analysis were performed using the 6,094,317 SNPs after filtering based on the criteria of minor allele frequency of >0.05 and missing data rate of 10% or less in the population. The kinship matrix (K) was generated using EMMAX (Kang et al., 2010), and the GWAS was performed using EMMAX with Q matrix. The modified Bonferroni correction was used to determine the genome-wide significance thresholds of the GWAS, based on a nominal level of α = 0.05, corresponding to a raw *P*-value of 8.2 × 10^−9^ or a –log10(*P*)-value of 8.08. Candidate genes were identified using the reference sequence *Sorghum bicolor* version 3.1.1, curated at Phytozome (Goodstein et al., 2012) 13 (https://phytozome-next.jgi.doe.gov/).

## Results

### Phenotyping

**Figure 1:**
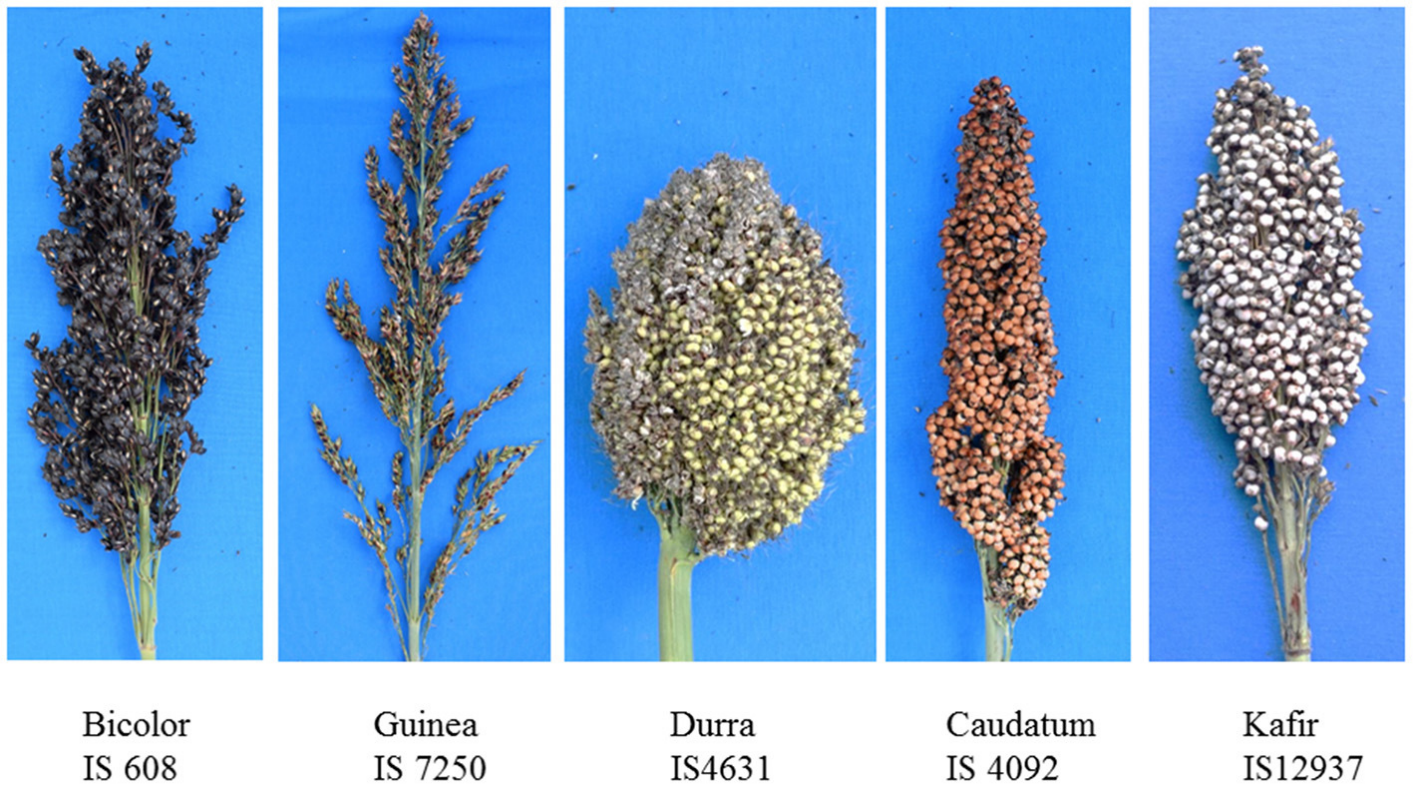
Panicle morphology of the five major races in the association mapping panel (Upadhyaya et al., 2009). IS 7250 has loose panicles, and IS 4631, IS 4092, and IS 12937 have compact panicles, whereas IS 608 has semi-compact panicles.

Panicle length and width were found to be correlated with Pearson's correlation coefficients ranging from 0.56 to 0.70 (significant at *P* < 0.001). Figure 1 shows variations in panicle morphology of the five primary sorghum races in the association mapping panel (Upadhyaya et al., 2009) from a field evaluation in Hainan in 2020. Based on the panicle compactness data from the Hainan 2020 environment, 64% of the MC accessions had compact panicles, 14% had semi-compact panicles, and 22% had loose panicles. In the 11 ICRISAT testing environments (Supplementary Table 1), panicle width was more variable across the environments than panicle length as measured by the coefficient of variation (CV). The CV for panicle length ranged from 0.27 to 0.39, with a mean of 0.33, while that for panicle width ranged from 0.21 to 0.61, with a mean of 0.48 (Table 1; refer to Supplementary Table 2 for variance). In contrast, irrigation in Environments 3 and 5 did affect panicle length and width compared to no irrigation in Environments 4 and 6 but not consistently. By comparing Environments 3 to 5, irrigation did not significantly affect the panicle length (*P* = 0.17) but decreased the panicle width by 1.42 cm on average (*P* = 0.0034). Between Environments 4 and 6, irrigation increased the panicle length by 1.9 cm on average (*P* = 0.0030) but decreased the panicle width by 1.85 cm on average (*P* = 0.000012). When panicle compactness was scored only as compact, semi-compact, and loose, panicle length and width were negatively correlated with panicle compactness with *r* = −0.40 and −0.27, respectively, in Environment 1 at ICRISAT, and both were significant at *P* < 0.001 (i.e., panicle compactness was only measured in Environment 1 at ICRISAT). Similarly, in the 2020 Hainan dataset, panicle length and width were negatively correlated with panicle compactness with *r* = −0.42, and −0.47, respectively, and both were also significant at *P* < 0.001. These results indicate that loose panicles tend to be longer and wider, and compact panicles are shorter and narrower. Using 100 seed weight data obtained from the studies by Upadhyaya et al. (unpublished) and Li et al. (unpublished), we found that seed weight was positively correlated with panicle compactness both at ICRISAT (*r* = 0.33; significant at *P* < 0.001) and Hainan (*r* = 0.31; significant at *P* < 0.001), indicating that loose panicles often carry smaller seeds and that compact panicles carry larger seeds. This may have contributed to the positive correlation between panicle compactness and seed weight per panicle (*r* = 0.23; significant at *P* < 0.01). Since the untransformed data were used in this study, heritability may not be as accurately estimated (Fusi et al., 2014), and small-effect QTLs may not be identified by GWAS (Goh and Yap, 2009). Nevertheless, variance, broad-sense heritability, and the Shapiro–Wilk normality test are presented in Supplementary Table 2.

**Table 1:** Coefficient of variation (CV) for panicle length and width in the 11 testing environments.

**Environment**	**1**	**2**	**3**	**4**	**5**	**6**	**7**	**8**	**9**	**10**	**11**
Panicle length	0.27	0.27	0.33	0.32	0.39	0.35	0.29	0.29	0.36	0.37	0.39
Panicle width	0.21	0.34	0.55	0.51	0.61	0.53	0.49	0.39	0.5	0.53	0.6

### Genome-Wide Association Study

For a trait to be mapped, the association had to be strong in multiple environments with multiple SNPs and reached the Bonferroni correction *P*-value of 8.2 × 10^−9^ or a –log(*P*) of 8.08, in at least two environments, except for recurving peduncles, which was evaluated only in one environment. Using these criteria, we identified 11 QTLs: one on chromosome 4 for panicle length/width ratio, two for peduncle recurving with one each on chromosomes 7 and 9, eight for panicle length and width, and one compactness colocalized with panicle length and width on chromosome 2 (Table 2; representing SNPs from each locus are presented in Supplementary Table 3). For the eight-panicle length and width QTLs, two were on chromosomes 1, 2, and 6, and one each was located on chromosomes 8 and 10 (Figure 2, Table 2, Supplementary Figures 2–9). Associations with *P*-values lower than the Bonferroni threshold were not observed in environments with a CV lower than the average, 0.33 and 0.48 for panicle length and width, respectively, except for panicle width in Environment 8 (Figure 2, Table 1, Supplementary Figures 2–9). *Pm 2-1* and *Pm 2-2* were both detected in the greatest number of environments with low *P*-values (Figure 2); *Pr 7-1* and *Pr 9-1* were associated with peduncle recurving with the lowest *P-*values (Supplementary Figure 9). We focused on these loci to identify candidate genes.

**Table 2:** Panicle morphology-related quantitative trait loci (QTLs) mapped in multiple environments.

**QTL**	Trait^*^	**Chromosome: position (bp)**	**Gene in or near QTL**	**No. of environment QTL detected**	**Colocalization with other QTL**	References^**^
*Pm 1-1*	PL, PW	1: 10423724–10464740	Sobic.001G132600	1 (PL), 4 (PW)		
*Pm 1-2*	PL, PW	1: 59803397–59808620	Sobic.001G311050	5 (PW)	*QPLEN1.7*	Hmon et al., 2013
*Pm 2-1*	PL, PW	2: 71879000–71902200	See Table 3	7 (PL), 5 (PW)		
*Pm 2-2*	PL, PW, PC	2: 73190000–73247000	See Table 3	7 (PL), 5 (PW), 1 (PC)		
*Pm 4-1*	PL/PW ratio	4: 8275699–8300275	Sobic.004G095300	2, 10, 11		
*Pm 6-1*	PL, PW	6: 32406706–32416278		1 (PL), 3 (PW)	*QPLEN6.6*	Reddy et al., 2013
*Pm 6-2*	PL, PW	6: 48330285–48349357	Sobic.006G115600	5 (PL), 3 (PW)	*QPLEN6.12*	Zou et al., 2012
*Pr 7-1*	PR	7: 8189476–8208789	Sobic.007G072600, Sobic.007G072800, Sobic.007G072901	1 (PR)		
*Pm 8-1*	PL, PW	8: 53337842–53434526	Promoter of Sobic.008G120200	3 (PL), 5 (PW)	*QPWTH8.1*	Zhou et al., 2019
*Pr 9-1*	PR	9: 4118798–4127062		1 (PR)		
*Pm 10-1*	PL, PW	10: 13724096–13790887		3 (PL), 2 (PW)	*QPTYP10.1*	Hmon et al., 2013

* *PL, panicle length; PW, panicle width; PC, panicle compactness; PR, peduncle recurving*.

#x0002A;*: *From the data cataloged by Mace et al. (2019)*.

**Figure 2:**
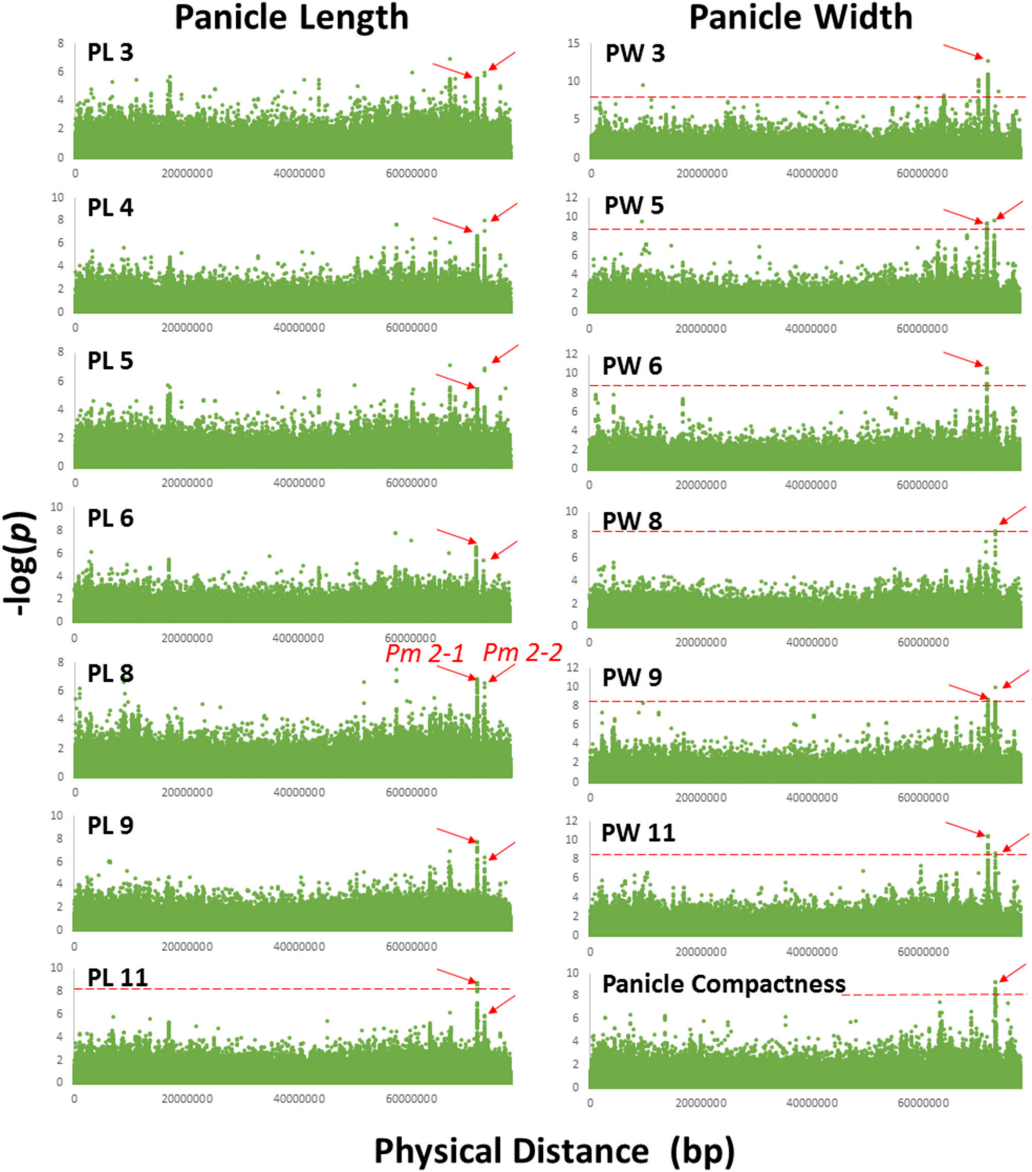
Manhattan plot of chromosome 2 showing *Pm 2-1* and *Pm 2-2* associated with panicle length and width in 7 and 6 out of 11 environments, respectively. Association with panicle compactness was identified in a separate environment. PL 3, 4, 5, 6, 8, 9, and 11 and PW 3, 5, 6, 8, 9, and 11 were the environments in which panicle length and width QTLs *Pm 2-1* and *Pm 2-2* were detected. The red dashed horizontal lines indicate the Bonferroni threshold *P*-value.

### Candidate Panicle Morphology Genes

To identify candidate panicle morphology-related genes, we examined genomic regions covered by each QTL in the *Sorghum bicolor* version 3.1.1 genome at Phytozome (Goodstein et al., 2012) 13 (https://phytozome-next.jgi.doe.gov/info/Sbicolor_v3_1_1). For the two peduncle QTLs, there were no protein-coding genes in the *Pr 9-1* locus (Table 2). However, *Pr 9-1* was 748 bp from the 5′ end of the Sobic.009G043600 coding region and 48 bp from the 5′ end of the Sobic.009G043500 coding region. Sobic.009G043600 encodes glutathione S-transferase 4, and Sobic.009G043500 encodes sulfite oxidase. There were three large genes (i.e., Sobic.007G072600, Sobic.007G072800, and Sobic.007G072901) and one small gene (i.e., Sobic.007G072700) in the *Pr 7-1* locus. Sobic.007G072600, Sobic.007G072800, and Sobic.007G072901 all encode F-box proteins. Sobic.007G072700 encodes an unknown protein specific to sorghum-based on a BLAST search.

**Figure 3:**
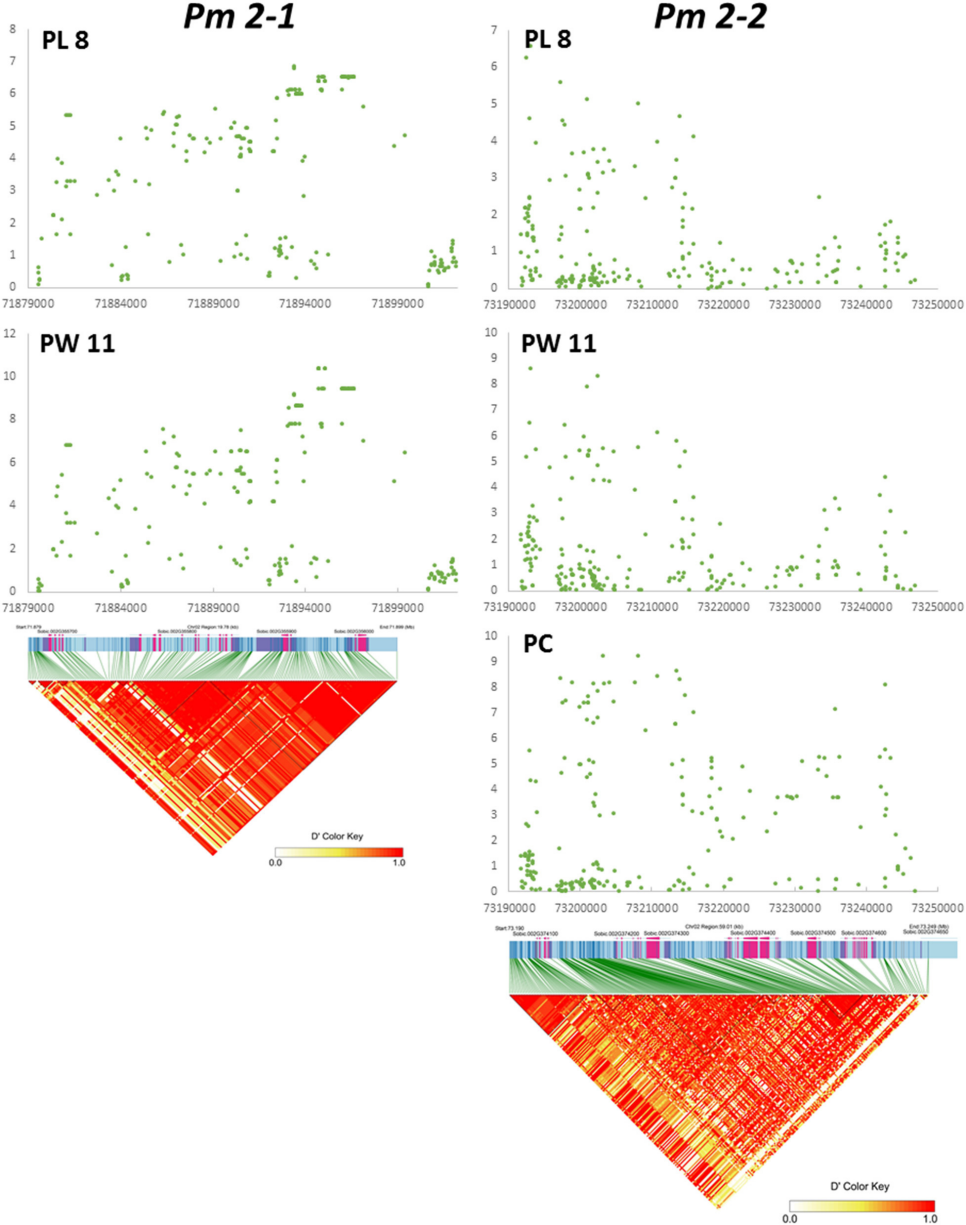
Detailed map of *Pm 2-1* and *Pm 2-2* loci. *X* and *Y* axes represent –log(*P*) and physical distance in bp, respectively. At the bottom of *Pm 2-1* and *Pm 2-2*, the panels are linkage disequilibrium (LD) plots aligned to the respective locus map.

We examined *Pm 2-1* and *Pm 2-2* loci in more detail. The genomic regions of the two loci are displayed in Figure 3 for panicle length and width from two testing environments and compactness from one. *Pm 2-1* included four genes, and *Pm 2-2* included six genes (Figure 3, Table 3). Each of the four genes (i.e., Sobic.002G355700, Sobic.002G355800, Sobic.002G355900, and Sobic.002G356000) in *Pm 2-1* resided in an LD block, except Sobic.002G355900, but in *Pm 2-2*, only Sobic.002G374400 was inside an LD block (Figure 3). Functional studies are necessary to identify the genes underlying each locus.

**Table 3:** Sorghum panicle morphology candidate genes in *Pm 2-1* and *Pm 2-2*.

**Sorghum gene ID**	**Annotation**
***Pm 2-1***	
Sobic.002G355700	Histone H3
Sobic.002G355800	Ca^2+^-binding protein
Sobic.002G355900	Lipid transfer protein
Sobic.002G356000	Lipid transfer protein
***Pm 2-2***	
Sobic.002G374100	Jasmonate ZIM domain-containing TIFY 10b
Sobic.002G374200	DNA-directed RNA polymerase
Sobic.002G374300	FAR1 transcription factor
Sobic.002G374400	*Erect panicle2* protein
Sobic.002G374500	Unknown protein
Sobic.002G374600	Beta-ketoacyl-ACP synthase

## Discussion

Our goal was to map major QTLs that are stable across environments and identify genes that can be used to improve economically important traits in sorghum and other species. In this study, we mapped nine panicle morphology QTLs, such as *Pm 2-1* and *Pm 2-2*, and two peduncle recurving QTLs, such as *Pr 7-1* and *Pr 9-1*. Neither *Pm 2-1, Pm 2-2, Pr 7-1*, and *Pr 9-1* were previously identified by other groups (Faye et al., 2019; Girma et al., 2019; Zhou et al., 2019; Olatoye et al., 2020), nor they were identified in 22 studies cataloged by Mace et al. (2019). The *Pr 7-1, Pm 2-1*, and *Pm 2-2* loci contained four, four, and six genes, respectively. The RNAseq data available at Phytozome (McCormick et al., 2018) may provide insight into their functions. In addition, LD can be used to identify candidate genes mapped by GWAS (Sulem et al., 2008). For the three genes in *Pr 7-1*, the highest expression of Sobic.007G072600 and Sobic.007G072901 was in both the peduncle and the panicle at the floral initiation stage, while the highest expression of Sobic.007G072800 was in the leaf sheath. Sobic.007G072700 was not expressed in the peduncles. Both Sobic.007G072600 and Sobic.007G072901 are good candidates in determining which gene in this locus causes recurving peduncles. Among the four genes in *Pm 2-1*, Sobic.002G355700 and Sobic.002G356000 were not expressed in peduncles and Sobic.002G355900 was almost exclusively expressed in dry seeds. The remaining Sobic.002G355800 was highly expressed in leaf sheaths, panicles, shoots, and stems, with slightly lower expression in peduncles, and resides inside an LD block (Figure 3). Therefore, Sobic.002G355800 is a candidate gene for the *Pm 2-1* locus. In the *Pm 2-2* locus, Sobic.002G374100 is co-expressed with genes in an anthesis stage-specific co-expression subnetwork with very low expression in peduncles; Sobic.002G374500 is not expressed in panicles or peduncles, and the highest expression of Sobic.002G374600 is in leaves and shoots. The remaining three genes (Sobic.002G374200, Sobic.002G374300, and Sobic.002G374400) were highly expressed in the panicles and peduncles. However, Sobic.002G374400 shares 66% identity and 77% similarity with *Erect Panicle2* (*EP2*) in indica rice and is the only gene inside an LD block (Figure 3). *EP2* regulates panicle erectness, panicle length, and grain size in rice (Zhu et al., 2010). The *EP2* mutants have shorter panicle length, more vascular bundles, and a thicker stem than that of wild-type plants, creating an erect panicle phenotype. *EP2* encodes a novel plant-specific protein localized to the endoplasmic reticulum with unknown function (Zhu et al., 2010) and is a candidate for the *Pm 2-2* locus. This is possible because panicle morphology regulation in both sorghum and rice may have similar mechanisms (Chen et al., 2015).

Previous studies have identified genes related to panicle/tassel morphology in the grasses. In maize, mutations in *Ramosa* produce a maize tassel resembling a loose sorghum panicle (Vollbrecht et al., 2005). *Ramosa1* transcription factor regulates long inflorescence branch architecture similarly in maize and sorghum but is absent in rice and heterochronically expressed in sorghum (Vollbrecht et al., 2005). Several panicle morphology-related genes have been identified in rice. A rice ncl-1, HT2A, and lin-41 (NHL)-domain-containing protein encoded by *FUWA* produces a more compact and erect panicle when the gene is mutated, and the mutant can be rescued by orthologs from sorghum and maize, indicating that the regulation of panicle morphology by this gene is evolutionarily conserved in rice, sorghum, and maize (Chen et al., 2015). The *OsLG1* gene product also regulates rice panicle compactness; its overexpression converts compact panicles to loose panicles. OsLG1 is an squamosa promoter-binding (SBP)-domain transcription factor that controls the development of rice ligules. The association analysis found that an SNP in the *OsLG1* regulatory region led to a compact panicle architecture in cultivated rice during rice domestication (Zhu et al., 2013). Another rice panicle morphology gene, *APO1*, encodes an F-box protein. The overexpression of *APO1* increases panicle branches and spikelets (Ikeda et al., 2007), whereas *APO1* mutation reduces the number of secondary branches by >90% and the total number of flowers by >70% (Ikeda et al., 2005). The abovementioned studies of *Ramosa* in maize and *FUWA* in rice, as well as the fact that the bulk of maize tassel and sorghum panicle developmental activities are shared (Leiboff and Hake, 2019), demonstrate similarities and differences in inflorescence development in maize, rice, and sorghum. Further studies are required to confirm whether the candidate genes identified in this study play a role in panicle morphology in sorghum and their possible effects on yield and related traits.

## Data Availability Statement

The datasets presented in this study can be found in online repositories. The names of the repository/repositories and accession number(s) can be found in the article/Supplementary Material.

## Author Contributions

HU, SS, CG, and RK phenotyped panicle length and width in the 11 environments at ICRISAT. LW, JZ, YL, and YZ performed phenotyping in Hainan 2017 and 2020. Y-HW scored panicle compactness in Hainan 2017 and 2020, as well as peduncle recurving in Hainan 2020 and wrote the manuscript. JL performed GWAS/LD analysis and normality test and calculated variance and broad-sense heritability. JL and Y-HW analyzed the GWAS results. All authors have read and approved the manuscript for publication.

## Funding

This study was supported by the National Natural Science Foundation of China (31971993), the Anhui Provincial Natural Science Fund (2008085MC73), the Anhui Provincial Key R&D Programs (202004b11020003), and the Key Project of Natural Science Research of the Anhui Provincial Education Department (KJ2019A0811).

## Conflict of Interest

The authors declare that the research was conducted in the absence of any commercial or financial relationships that could be construed as a potential conflict of interest.

## Publisher's Note

All claims expressed in this article are solely those of the authors and do not necessarily represent those of their affiliated organizations, or those of the publisher, the editors and the reviewers. Any product that may be evaluated in this article, or claim that may be made by its manufacturer, is not guaranteed or endorsed by the publisher.

## Supplementary Material

Supplementary Figure 1 Distribution of 489,339 SNPs used in population structure analysis.

Supplementary Figure 2 Manhattan plot of chromosome 1 showing *Pm* 1-1 associated with panicle length (PL) and panicle width (PW) (red arrow). Numbers after PL or PW indicate different testing environments. Red dashed line indicates Bonferroni threshold *P*-value.

Supplementary Figure 3 Manhattan plot of chromosome 1 showing *Pm 1-2* associated with panicle length (PL) and panicle width (PW) (red arrow). Numbers after PL or PW indicate different testing environments. Red dashed line indicates Bonferroni threshold *P*-value.

Supplementary Figure 4 Manhattan plot of chromosome 4 showing *Pm 4-1* associated with panicle length and width ratio (red arrow). Numbers after LW indicate different testing environments. Red dashed line indicates Bonferroni threshold *P*-value.

Supplementary Figure 5 Manhattan plot of chromosome 6 showing Pm 6-i associated with panicle length (PL) and panicle width (PW) (red arrow). Numbers after PL or PW indicate different testing environments. Red dashed line indicates Bonferroni threshold *P*-value.

Supplementary Figure 6 Manhattan plot of chromosome 6 showing *Pm 6-2* associated with panicle length (PL) and panicle width (PW) (red arrow). Numbers after PL or PW indicate different testing environments. Red dashed line indicates Bonferroni threshold *P-*value.

Supplementary Figure 7 Manhattan plot of chromosome 8 showing *Pm 8-1* associated with panicle length (PL) and panicle width (PW) (red arrow). Numbers after PL or PW indicate different testing environments. Red dashed line indicates Bonferroni threshold *P*-value.

Supplementary Figure 8 Manhattan plot of chromosome 10 showing *Pm 10-1* associated with panicle length (PL) and panicle width (PW) (red arrow). Numbers after PL or PW indicate different testing environments. Red dashed line indicates Bonferroni threshold *P-*value.

Supplementary Figure 9 Manhattan plot of chromosome 7 showing *Pr 7-1* (top) and 9 showing *Pr 9-1* (bottom) associated with peduncle recurving (red arrow). Red dashed line indicates Bonferroni threshold *P*-value.

Supplementary Table 1 Environment and traits evaluated in this study. ^*^PRi-postrainy season with irrigation; PR-postrainy season; R-rainy season. ^**^PC, panicle compactness; PL, panicle length; PR, peduncle recurving; PW, panicle width.

Supplementary Table 2 Variance, heritability and normality for panicle length and width in the 11 testing environments.

Supplementary Table 3 SNPs associated with panicle morphological traits in sorghum.
