# Supplementary figures and images for: Genome-Wide Association Mapping Identifies Novel Panicle Morphology Loci and Candidate Genes in Sorghum
Source: Front Plant Sci. 2021 Oct 5;12:743838. doi: 10.3389/fpls.2021.743838 (PMC8525895; doi:10.3389/fpls.2021.743838)

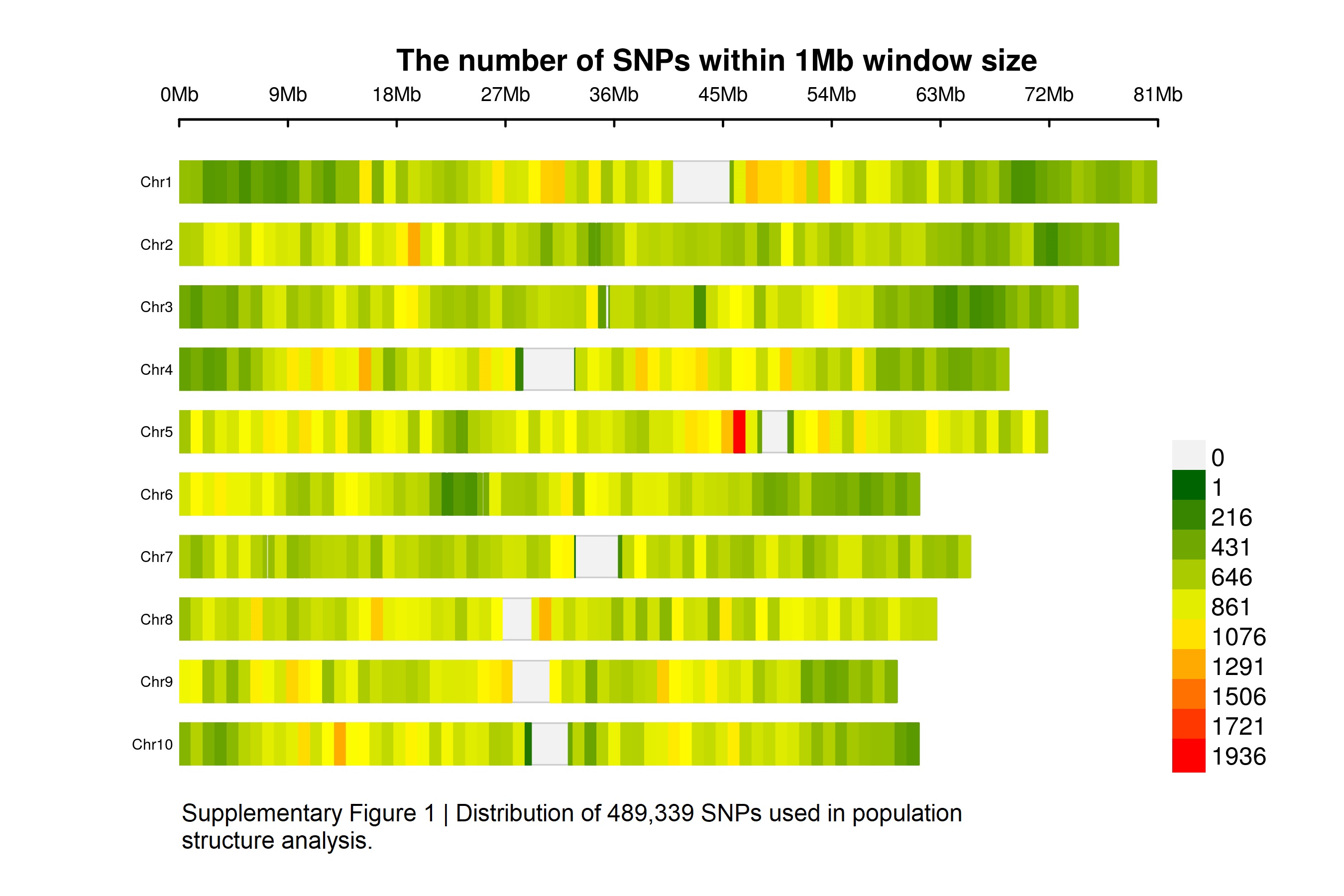

Supplement: Supplementary Figure 1 — Distribution of 489,339 SNPs used in population structure analysis. [file Image_1.jpg]

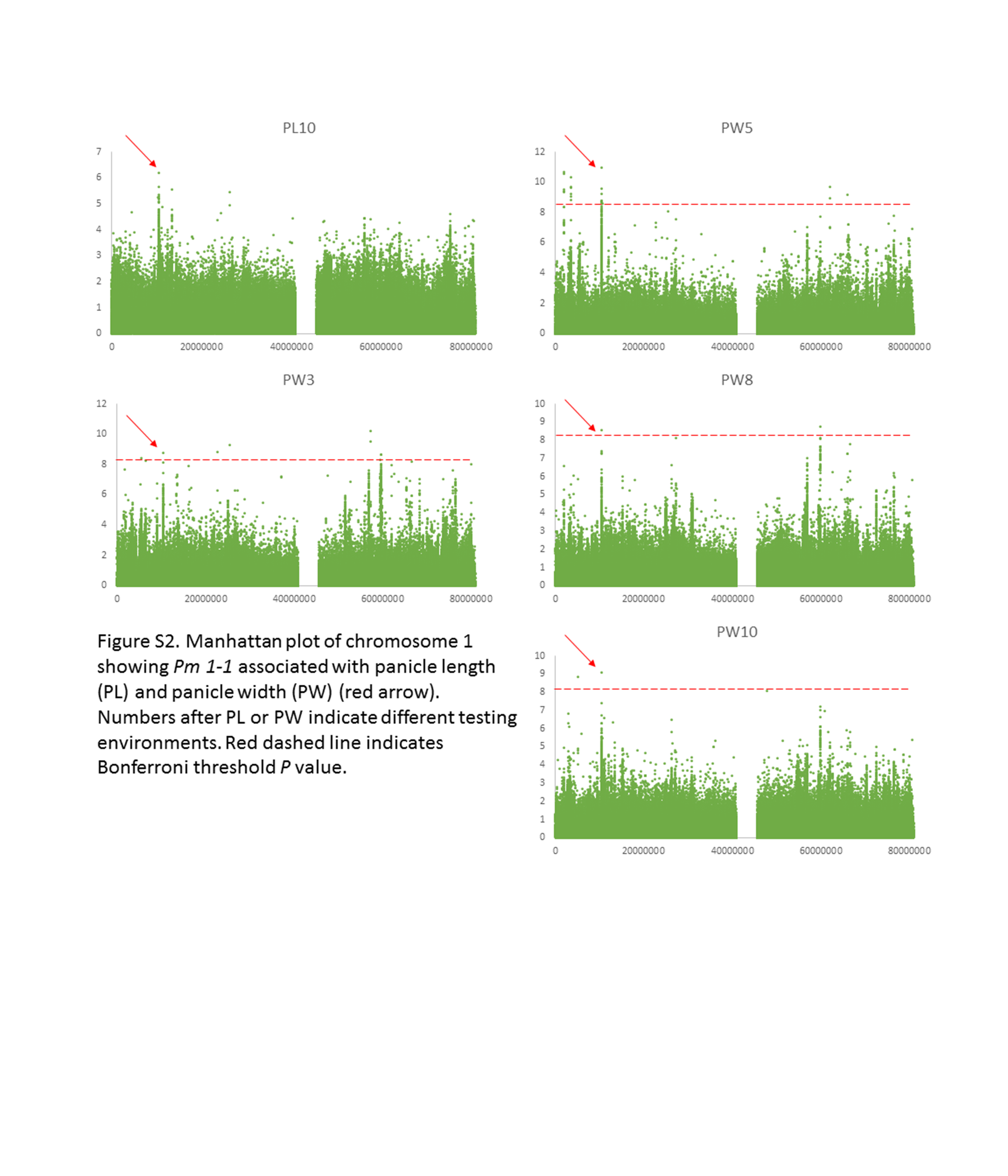

Supplement: Supplementary Figure 2 — Manhattan plot of chromosome 1 showing Pm 1-1 associated with panicle length (PL) and panicle width (PW) (red arrow). Numbers after PL or PW indicate different testing environments. Red dashed line indicates Bonferroni threshold P-value. [file Image_2.tif]

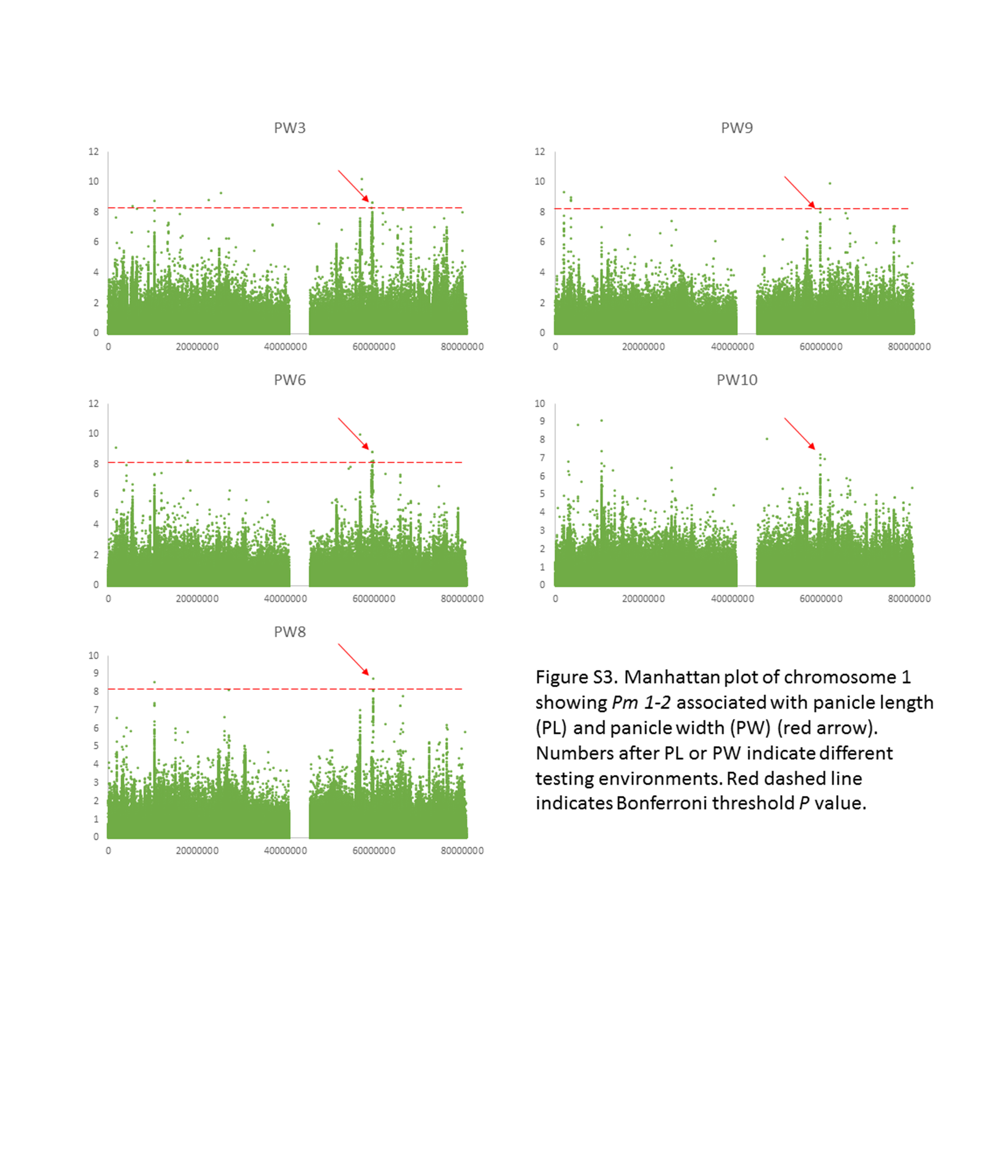

Supplement: Supplementary Figure 3 — Manhattan plot of chromosome 1 showing Pm 1-2 associated with panicle length (PL) and panicle width (PW) (red arrow). Numbers after PL or PW indicate different testing environments. Red dashed line indicates Bonferroni threshold P-value. [file Image_3.tif]

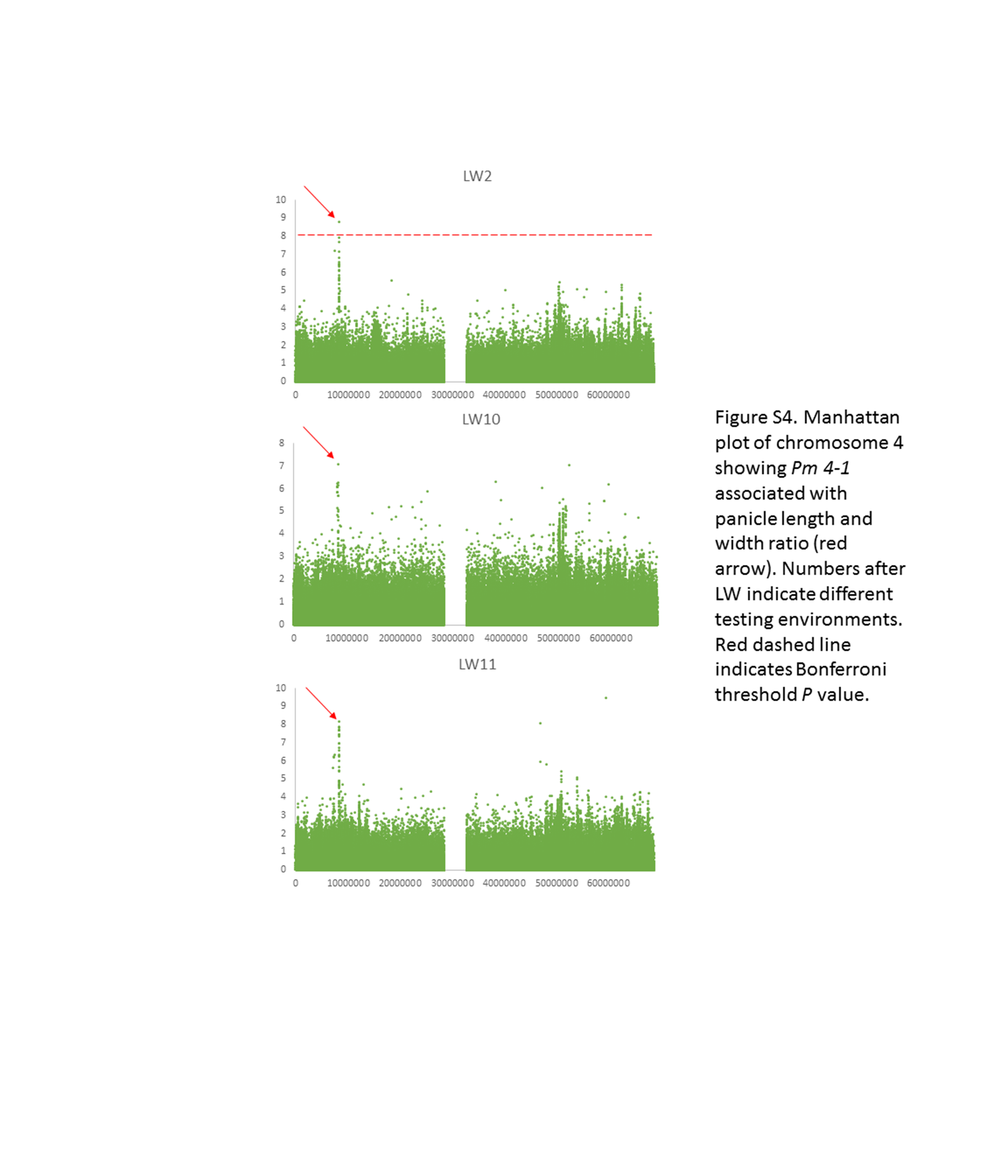

Supplement: Supplementary Figure 4 — Manhattan plot of chromosome 4 showing Pm 4-1 associated with panicle length and width ratio (red arrow). Numbers after LW indicate different testing environments. Red dashed line indicates Bonferroni threshold P-value. [file Image_4.tif]

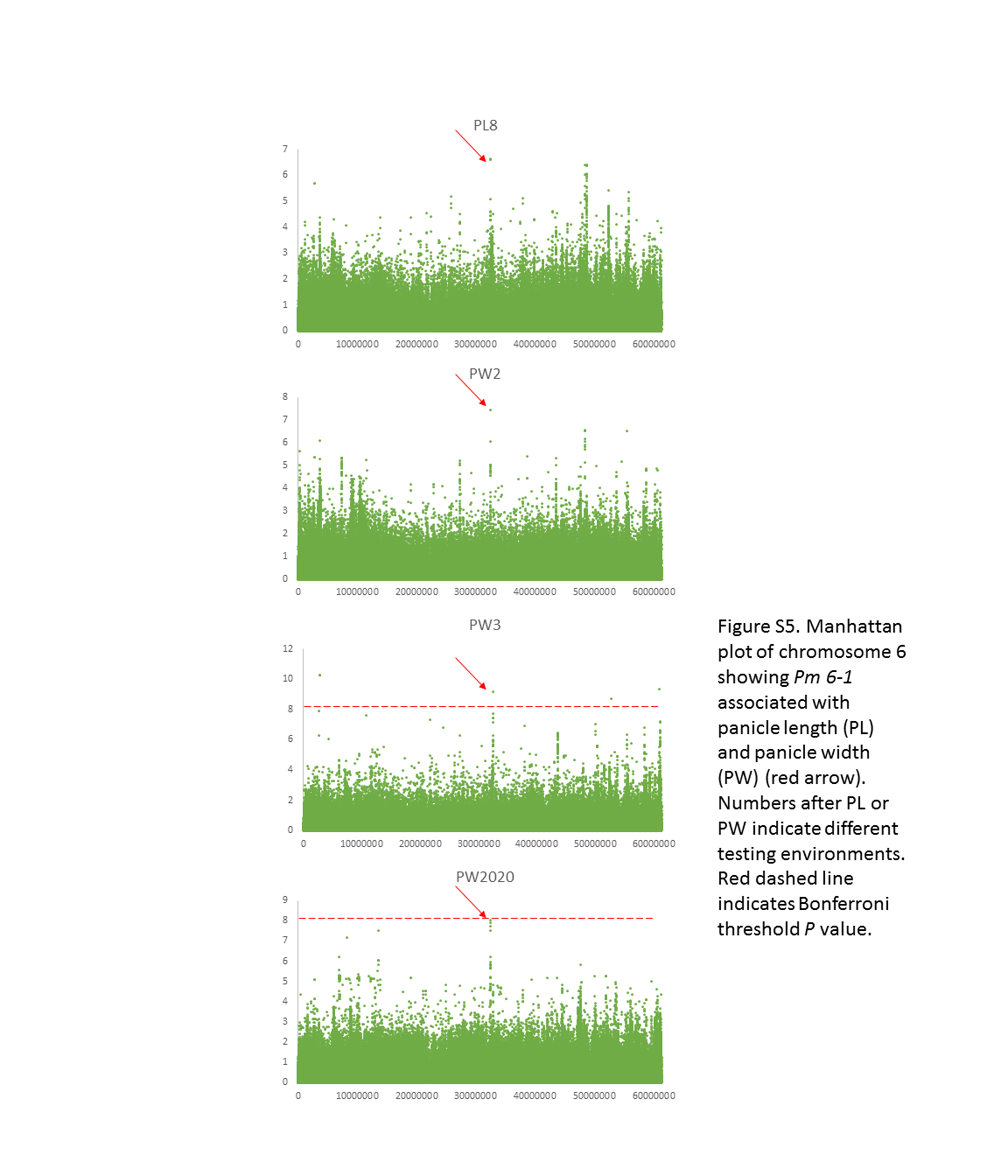

Supplement: Supplementary Figure 5 — Manhattan plot of chromosome 6 showing Pm 6-i associated with panicle length (PL) and panicle width (PW) (red arrow). Numbers after PL or PW indicate different testing environments. Red dashed line indicates Bonferroni threshold P-value. [file Image_5.tif]

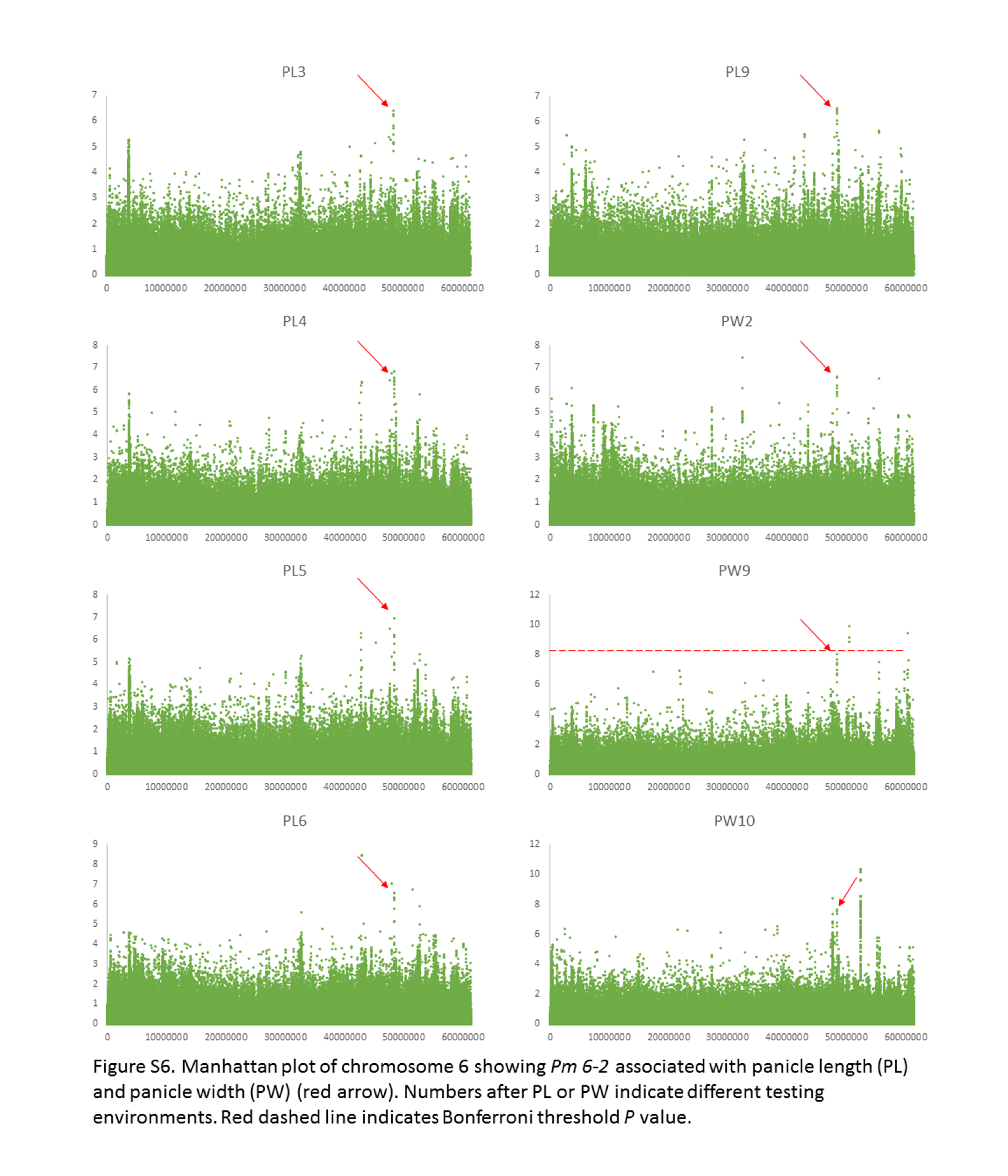

Supplement: Supplementary Figure 6 — Manhattan plot of chromosome 6 showing Pm 6-2 associated with panicle length (PL) and panicle width (PW) (red arrow). Numbers after PL or PW indicate different testing environments. Red dashed line indicates Bonferroni threshold P-value. [file Image_6.tif]

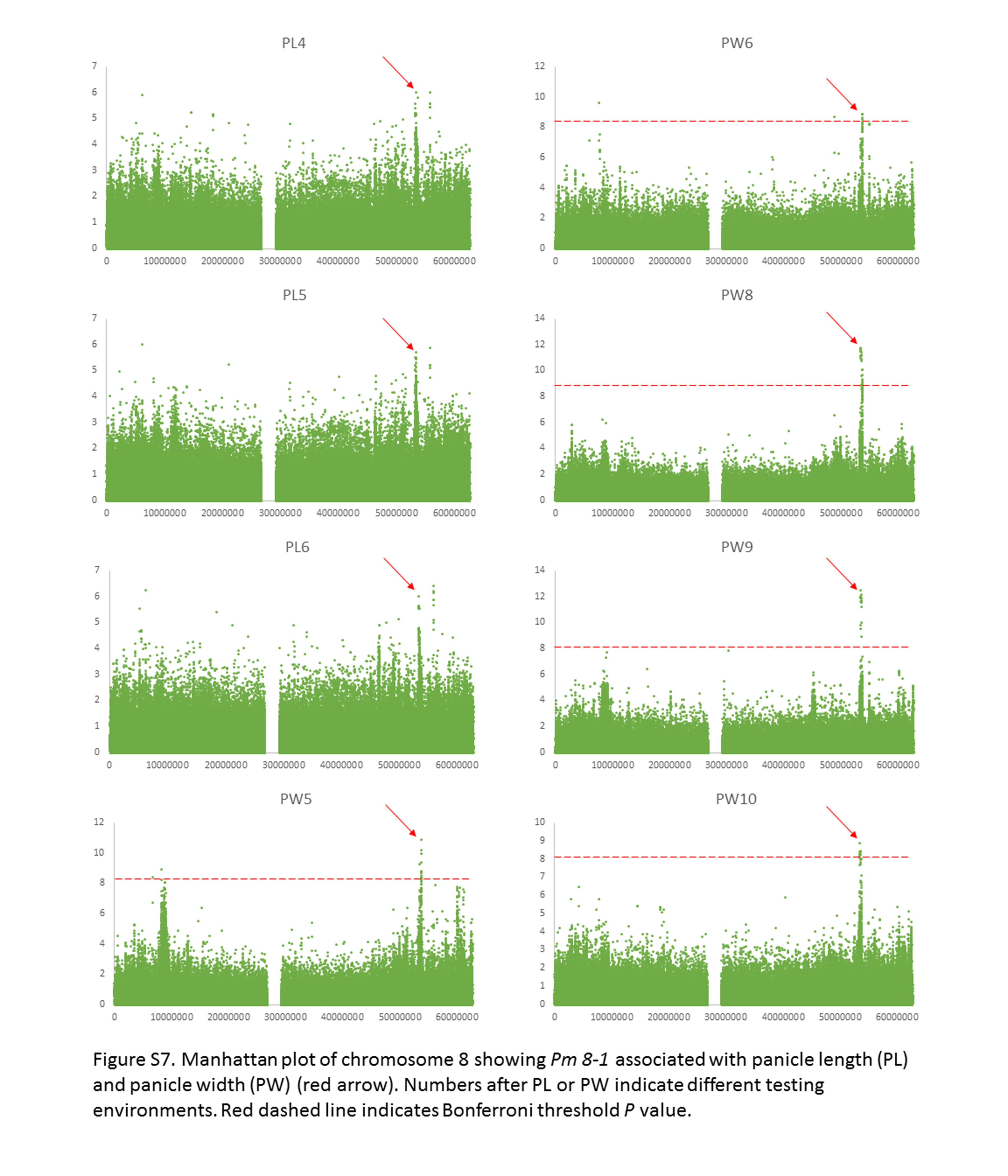

Supplement: Supplementary Figure 7 — Manhattan plot of chromosome 8 showing Pm 8-1 associated with panicle length (PL) and panicle width (PW) (red arrow). Numbers after PL or PW indicate different testing environments. Red dashed line indicates Bonferroni threshold P-value. [file Image_7.tif]

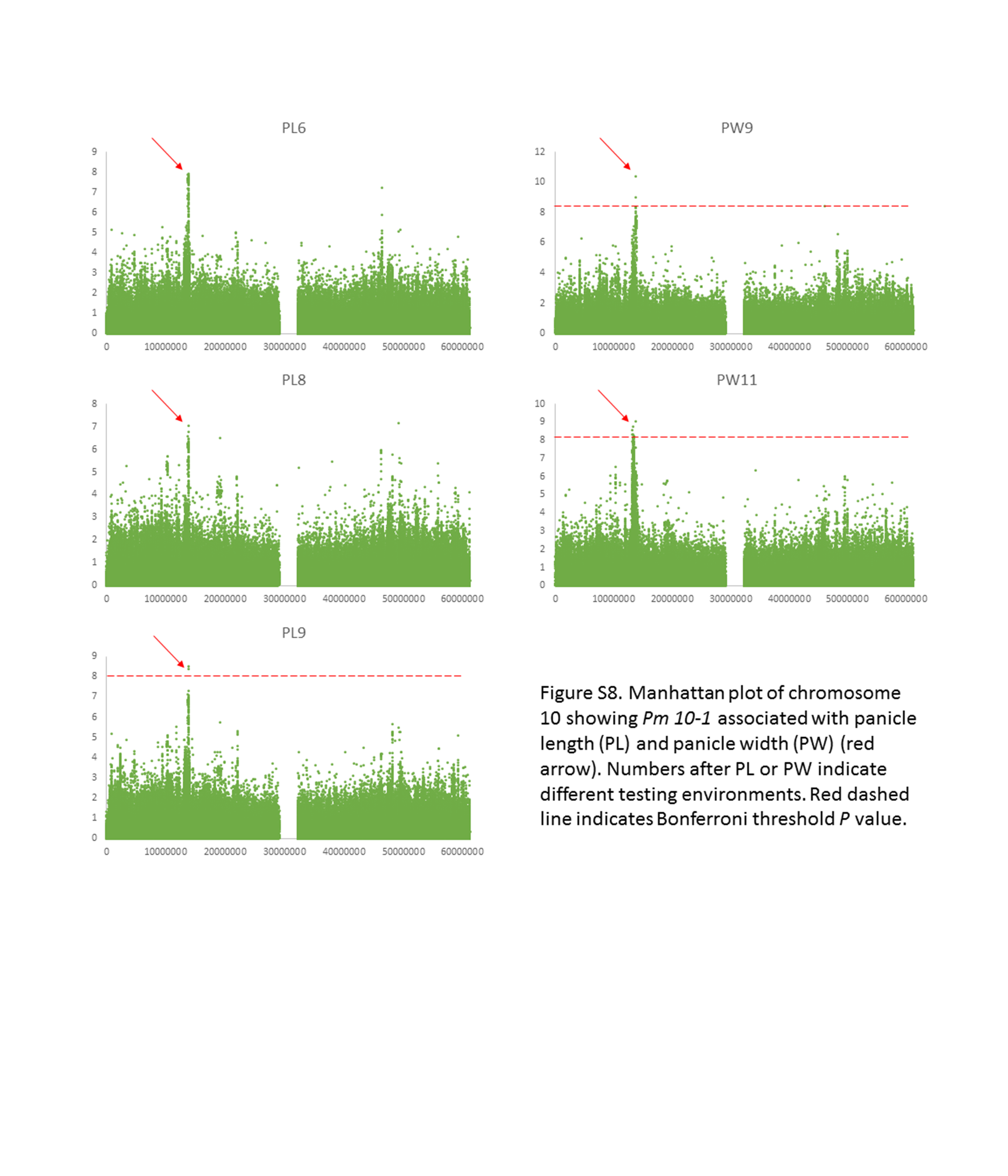

Supplement: Supplementary Figure 8 — Manhattan plot of chromosome 10 showing Pm 10-1 associated with panicle length (PL) and panicle width (PW) (red arrow). Numbers after PL or PW indicate different testing environments. Red dashed line indicates Bonferroni threshold P-value. [file Image_8.tif]

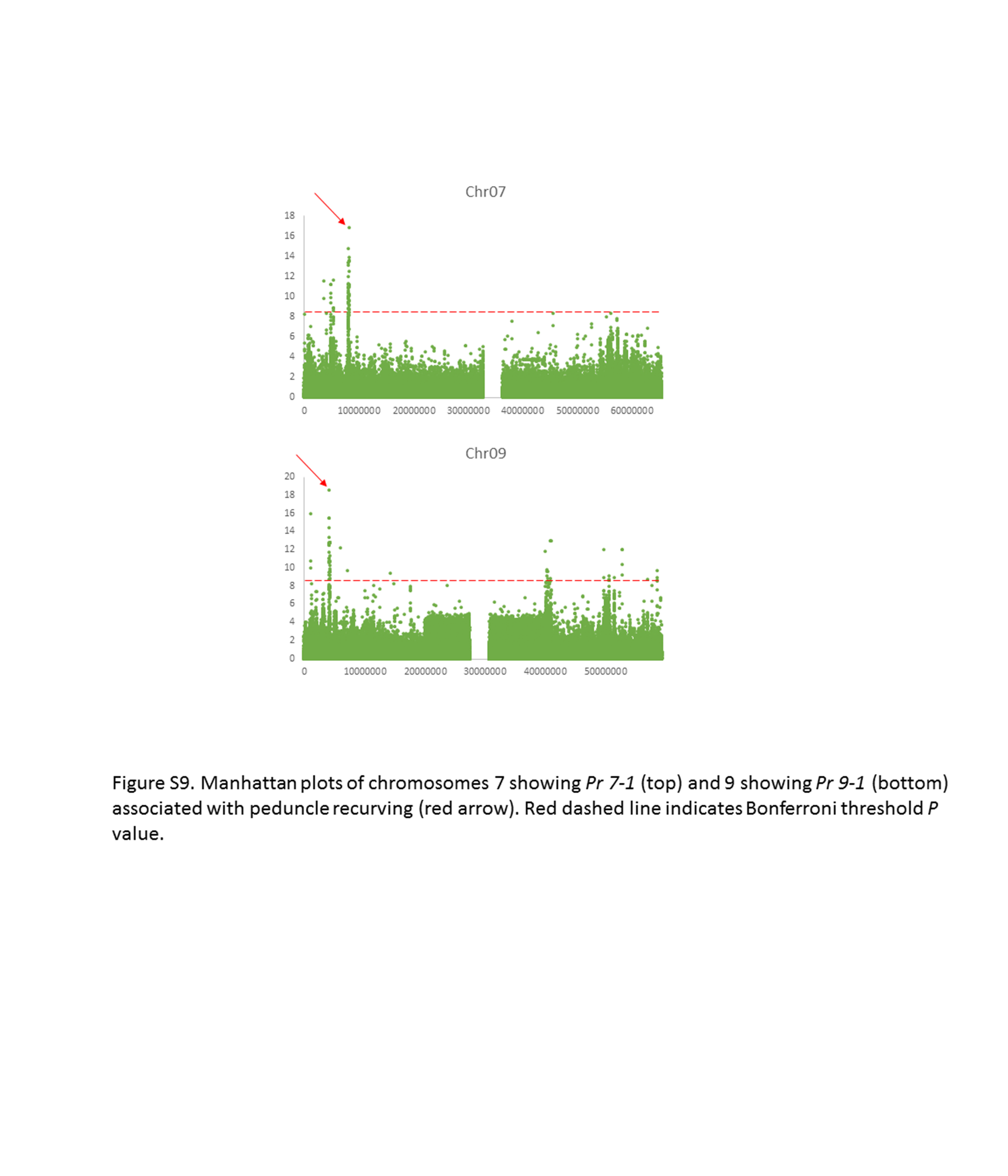

Supplement: Supplementary Figure 9 — Manhattan plot of chromosome 7 showing Pr 7-1 (top) and 9 showing Pr 9-1 (bottom) associated with peduncle recurving (red arrow). Red dashed line indicates Bonferroni threshold P-value. [file Image_9.tif]
