# Supplementary material for: Genome-Wide Association Mapping Identifies Novel Panicle Morphology Loci and Candidate Genes in Sorghum
Source: Front Plant Sci. 2021 Oct 5;12:743838. doi: 10.3389/fpls.2021.743838 (PMC8525895; doi:10.3389/fpls.2021.743838)
Supplement: Supplementary Table 1 — Environment and traits evaluated in this study. *PRi-postrainy season with irrigation; PR-postrainy season; R-rainy season. **PC, panicle compactness; PL, panicle length; PR, peduncle recurving; PW, panicle width. [file Data_Sheet_1.docx]

Table S1. Environment and traits evaluated in this study.

| ICRISAT, India |  |  |  |  |  |  |  |  |  |  |  |
| --- | --- | --- | --- | --- | --- | --- | --- | --- | --- | --- | --- |
| Environment code | 1 | 2 | 3 | 4 | 5 | 6 | 7 | 8 | 9 | 10 | 11 |
| Environment * | 2006 PRi | 2009 PRi | 2010 PRi | 2011 PRi | 2010 PR | 2011 PR | 2007 R | 2008 R | 2009 R | 2010 R | 2011 R |
| Traits measured** | PL, PW | PL, PW | PL, PW | PL, PW | PL, PW | PL, PW | PL, PW | PL, PW | PL, PW | PL, PW | PL, PW |
|  |  |  |  |  |  |  |  |  |  |  |  |
| Hainan, China |  |  |  |  |  |  |  |  |  |  |  |
| Environment code | 2017 | 2020 |  |  |  |  |  |  |  |  |  |
| Environment | Nov-April | Nov-April |  |  |  |  |  |  |  |  |  |
| Traits measured | PC | PC, PR, PL, PW |  |  |  |  |  |  |  |  |  |

Notes: *PRi-postrainy season with irrigation; PR-postrainy season; R-rainy season.

**PC-panicle compactness; PL-panicle length; PR-peduncle recurving; PW-panicle width.

Table S2. Variance, heritability and normality for panicle length and width in the 11 testing environments.

| Environment | 1 | 2 | 3 | 4 | 5 | 6 | 7 | 8 | 9 | 10 | 11 |
| --- | --- | --- | --- | --- | --- | --- | --- | --- | --- | --- | --- |
| Panicle length |  |  |  |  |  |  |  |  |  |  |  |
| Variance | 6.90 | 29.90 | 43.37 | 49.54 | 49.55 | 39.29 | 29.70 | 31.66 | 40.91 | 52.14 | 54.28 |
| Broad-sense heritability | 0.27 | 0.88 | 0.94 | 0.94 | 0.95 | 0.90 | 0.71 | 0.77 | 0.93 | 0.97 | 0.94 |
| Shapiro-Wilk normality test (*P*) | <0.05 | 0.12 | 0.21 | <0.05 | 0.14 | <0.05 | <0.05 | <0.05 | <0.05 | 0.19 | <0.05 |
|  |  |  |  |  |  |  |  |  |  |  |  |
| Panicle width |  |  |  |  |  |  |  |  |  |  |  |
| Variance | 0.02 | 3.72 | 22.39 | 23.89 | 37.52 | 17.13 | 6.21 | 7.79 | 8.29 | 15.00 | 38.18 |
| Broad-sense heritability | 0 | 0.71 | 0.96 | 0.89 | 0.97 | 0.88 | 0.59 | 0.53 | 0.76 | 0.94 | 0.94 |
| Shapiro-Wilk normality test (*P*) | <0.05 | <0.05 | <0.05 | <0.05 | <0.05 | <0.05 | <0.05 | <0.05 | <0.05 | <0.05 | <0.05 |

Table S3. SNPs associated with panicle morphological traits in sorghum.

| QTL | Trait | Associating SNPs | -log(*P*) | Environment |
| --- | --- | --- | --- | --- |
| *Pm 1-1* | PL, PW | 1:10431107  1:10464740 | 5.27  9.08 | 10  10 |
| *Pm 1-2* | PW | 1:59803397 | 8.76 | 8 |
| *Pm 2-1* | PL, PW | 2:71894701  2:71894701 | 8.72  10.38 | 11  11 |
| *Pm 2-2* | PL, PW, PC | 2:73193040  2:73202531  2:73203231 | 7.95  9.91  9.20 | 4  9  Hainan 2020 |
| *Pm 4-1* | PL/PW ratio | 4:8295990 | 8.79 | 2 |
| *Pm 6-1* | PL, PW | 6:32410039  6:32416278 | 6.64  9.16 | 8  3 |
| *Pm 6-2* | PL, PW | 6:48347933  6:48345655 | 6.95  8.02 | 5  9 |
| *Pr 7-1* | PR | 7:8196564 | 16.79 | Hainan 2020 |
| *Pm 8-1* | PL, PW | 8:53340846  8:53434526 | 5.72  8.87 | 5  10 |
| *Pr 9-1* | PR | 9:4123616 | 18.51 | Hainan 2020 |
| *Pm 10-1* | PL, PW | 10:13745149  10:13775581 | 8.39  10.37 | 9  9 |
